# Health diplomacy training, pedagogical approaches, and skills assessment: a scoping review

**DOI:** 10.3389/fpubh.2025.1729728

**Published:** 2025-12-16

**Authors:** Ashish Joshi, Laura Magana, Niharika Jha, Robert Otok, Ntuli Angyelile Kapologwe, Luís de Almeida Sampaio, Erica Kastrup, Woldekidan Amde, Rajendra Surenthirakumaran, Ayman El-Mohandes, Rodrigo Reis, So Yoon Kim, Catherine Kane, Tim K. Mackey, William Yotive, Henrique Barros, Ramune Kaledine, Josep Figueras, Mathew D. Brown

**Affiliations:** 1 School of Public Health, University of Memphis, Memphis, TN, United States; 2 Association of Schools and Programs of Public Health (ASPPH), Washington, DC, United States; 3 Association of Schools of Public Health in the European Region (ASPHER), Brussels, Belgium; 4 East, Central and Southern Africa Health Community (ECSA-HC), Arusha, Tanzania; 5 Universidade de Coimbra, Coimbra, Portugal; 6 CRIS Center for International Relations in Health of the Oswaldo Cruz Foundation, Rio de Janeiro, Brazil; 7 School of Public Health, University of the Western Cape, Bellville, South Africa; 8 Department of Community Medicine, University of Jaffna, Jaffna, Sri Lanka; 9 CUNY Graduate School of Public Health and Health Policy, New York, NY, United States; 10 People Health and Place Unit, Prevention Research Center, Washington University in St. Louis, St. Louis, MO, United States; 11 Department of Medical Law and Ethics, Yonsei University, Seoul, Republic of Korea; 12 Independent Researcher, Geneva, Switzerland; 13 Global Health Program, Department of Anthropology, UC San Diego, San Diego, CA, United States; 14 Institute of Public Health, University of Porto, Porto, Portugal; 15 Lithuanian University of Health Sciences, Kaunas, Lithuania; 16 European Observatory on Health Systems and Policies, Brussels, Belgium; 17 Global Health Policy and Data Institute, UC San Diego, San Diego, CA, United States; 18 World Federation of United Nations Associations, Geneva, Switzerland

**Keywords:** health diplomacy, public health diplomacy, global health diplomacy, competency based education, health diplomacy training

## Abstract

**Background:**

Health diplomacy is gaining increasing importance as an approach in addressing domestic and global health challenges, yet educational programs that prepare future practitioners remain underdeveloped in addressing skills core to this domain of public health practice. Training in health diplomacy is critical for building interdisciplinary competencies needed to navigate increasingly complex negotiations, cross-cultural engagements, and policy influence. Competency based education in global health, widely accepted by the health professions education community, is a framework for training health professionals that focuses on observable, measurable skills and knowledge needed to meet specific health needs and improve global health outcomes.

**Objectives:**

This study mapped the literature on health diplomacy education, examining curricula, training approaches, skill development, and evaluation practices, with a focus on their implications for public health diplomacy.

**Methods:**

Establishing scoping review and inclusion methodology, this study conducted a systematic search and screening of relevant literature. Eligible documents included peer-reviewed articles, frameworks, and reports describing curricula, training initiatives, and educational models in health diplomacy. We extracted and synthesized data using descriptive statistics to map training types, audiences, and competencies, alongside narrative synthesis to identify pedagogical strategies, evaluation methods, gaps, and formulate key insights.

**Results:**

We included eight training initiatives and frameworks published between 2017 and 2025. Programs ranged from short-term simulations and workshops to semester-long academic curricula, flexible competency frameworks, and career-long professional pathways. Training was predominantly designed for students and early-career professionals, but also included experienced diplomatic practitioners such as health attachés. Delivery was largely in-person, with increasing adoption of blended and adaptable models. Common pedagogical methods included simulation-based experiential learning, problem- and competency-based approaches, peer-to-peer learning, and reflexive or decolonial pedagogy. Core competencies emphasized negotiation, diplomacy, cross-cultural communication, leadership, policy analysis, and crisis management. Evaluation methods were mostly short-term and self-reported, with limited evidence of long-term or institutional outcomes.

**Conclusion:**

Health diplomacy education is key in strengthening the practice of public health diplomacy by equipping learners with essential skills in negotiation, leadership, cultural competency, and communication skills. However, current training initiatives remain fragmented, inequitable, and under-evaluated.

## 1 Background

Understanding the intersection of health, domestic and foreign policy, and international relations is becoming increasingly critical to resolving local and global challenges such as pandemics, climate change, and migration. Each creates complex health, social, economic, and geopolitical challenges (1) that demand coordinated responses, frequently traversing borders, and involving nation states, international institutions, and the public to resolve. All such multidimensional challenges impact the public’s health in overt and nuanced ways. Addressing these challenges effectively requires integrating public health into all policy areas through a, “Health in All Policies” approach. Public health diplomacy presents a model that addresses complex challenges impacting societies, economies, and health by harmonizing, local action, policy with health development goals. Employing the tools of public health diplomacy can foster collaboration across governments, multilateral organizations, non-governmental organizations (NGOs), academia, research and the private sector, towards advancing global health priorities while also helping to adapt solutions to local communities, promoting shared development, and security goals (2). This convergence, commonly referred to as global health diplomacy (GHD), which in the opinion of the authors should not be to the exclusion of local practice and action, involves negotiation, advocacy, and governance processes that shape health policy across borders (2, 3). For practitioners to be effective, engagements in GHD require not only technical expertise in multiple public health domains, but also diplomatic competencies, including negotiation, policy analysis, cross-cultural communication, and leadership (4, 5). As health remains increasingly embedded in foreign policy agendas, there is a growing need to prepare professionals who can operate effectively at this critical interface between domestic and global arenas.

In response, various training initiatives and educational programs have emerged to develop GHD capacities targeting various actors. These range from short-term simulations and workshops, to structured academic curricula and professional development pathways (6–8). Such programs aim to equip students, policymakers, diplomats, and health professionals with the knowledge, skills and competencies required to navigate multilateral negotiations and governance processes. Curricula must provide the foundational knowledge, skills and attitudes, along with developing the measurable and observable behaviors (competencies) to navigate complexity, build consensus and make decisions. However, despite this proliferation, the evidence base on the scope, design, and outcomes of health diplomacy education remains fragmented. Many programs appear to operate in isolation, employ heterogeneous pedagogical approaches, and use inconsistent evaluation methods.

While previous studies have conceptualized the role of health diplomacy in global governance (3, 9), few studies have systematically examined the training programs’ structure, competencies emphasized, and assess effectiveness. Addressing these gaps is essential for building a more coherent approach to GHD education and training, to ensure future practitioners are equipped to advance public health goals through more effective diplomatic engagement and action.

The objectives of this scoping review are:

- To identify existing curricula, training programs, and educational initiatives in health diplomacy available globally;
- To examine the pedagogical approaches, skill domains, and competencies emphasized;
- To highlight gaps for future capacity-building in health diplomacy education.

## 2 Methodology

This scoping review follows the methodological framework proposed by Arksey and O’Malley (10), which provides a structured approach to mapping existing evidence on a given topic. The research team conducted the literature search and screening process between July 2025 and September 2025.

### 2.1 Identifying the research question

The following research question guided the review: What is the current landscape of health diplomacy education, including curricula, training approaches, learning outcomes, competencies, and evaluation methods available globally across peer-reviewed literature?

### 2.2 Identifying relevant studies

The research team conducted a systematic search to identify studies on health diplomacy education, with eligibility criteria defined *a priori* to ensure consistency and transparency in study selection. Inclusion criteria were broad and encompassed peer-reviewed articles, conference papers, reports, policy documents, and relevant gray literature. Studies were eligible if they described curricula, training modules, workshops, or structured skill-development programs in health diplomacy or global health diplomacy. The team also included publications that focused on competencies, training frameworks, or evaluation of health diplomacy programs. To ensure contemporary relevance, the team only included studies published between 2015 and 2025, and included documents only published in English. Exclusion criteria applied to articles that were unrelated to education or training in health diplomacy, as well as purely theoretical or conceptual papers that did not reference specific curricula, training, or skill-development activities.

The information sources for this review included two main electronic databases: PubMed, to capture peer-reviewed biomedical and health-related literature, and Google, to ensure coverage of gray literature, policy reports, and less traditional academic outputs (e.g., conference proceedings, institutional reports, and training manuals). The team searched the PubMed database using separate targeted searches for each component (e.g., “global health diplomacy AND curriculum,” “public health diplomacy AND training”), as shown in Supplementary Table S1. This approach was adopted to improve the specificity of results after an initial combined search yielded a large number of irrelevant records due to the broad and interdisciplinary use of these terms.

### 2.3 Study selection

**Figure 1:**
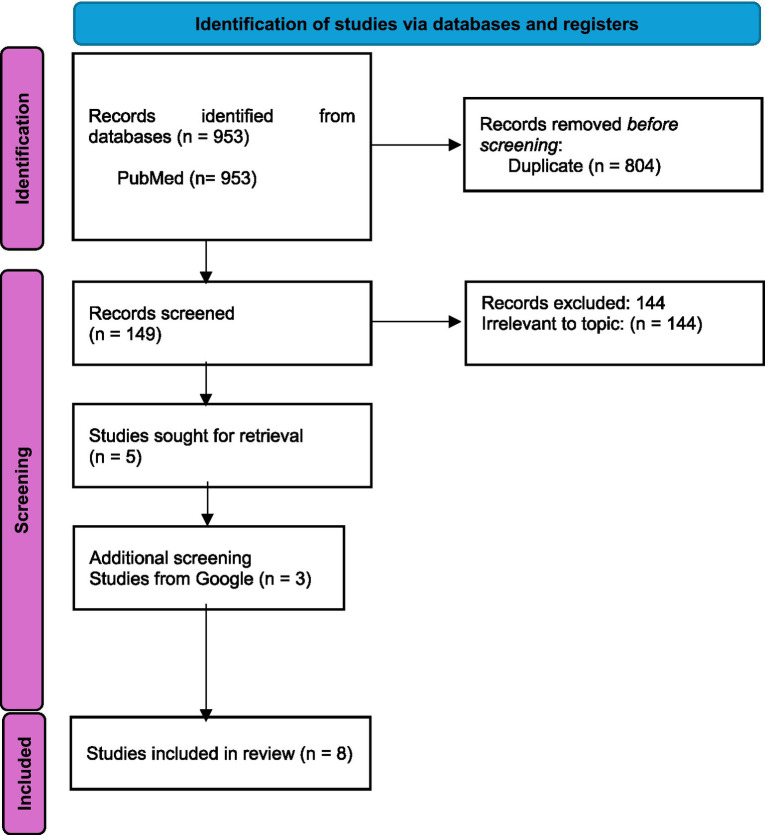
Flowchart of study identification, screening, and inclusion following PRISMA guidelines.

The research team imported the retrieved citations into a reference management software (Zotero). The search returned *n* = 953 records. After checking for duplicates (*n* = 804) a total of 149 documents were included for titles and abstracts screening. Two reviewers (NJ and AJ) independently screened (*n* = 149) abstracts. A total of 144 records were excluded after screening titles and abstracts for relevance, leaving five articles for full-text review and inclusion. To ensure comprehensive coverage, we also conducted a hand search through Google, which identified three additional relevant studies that met the eligibility criteria. A total of eight studies were included in this scoping review. Any discrepancies were resolved through consensus. The PRISMA flow diagram (Figure 1) reflects the study selection process.

### 2.4 Charting the data

The team developed a standardized data charting form in excel to ensure consistency in extracting and organizing information from the included studies. The charting process captured key characteristics (Table 1) of each publication, including author, year of publication, country, and type of document. The form recorded details of the training initiative, such as the type of initiative (e.g., curriculum, training program, workshop, short course, or academic program), the target audience (students, professionals, diplomats, or health workers), and the duration and delivery mode of the intervention. We extracted information on the pedagogical methods employed (such as simulation, problem-based learning, or peer-to-peer approaches), alongside the competencies and skills addressed, including domains such as negotiation, policy analysis, and cross-cultural communication. Finally, the charting process documented the evaluation methods used in each study and the reported outcomes, providing a comprehensive framework for mapping and synthesizing evidence across diverse training initiatives.

**Table 1:** Data extraction for scoping review.

Study ID	Title	Authors and year	Type of initiative	Target audience	Duration	Delivery mode	Pedagogical methods	Competencies/skills addressed	Evaluation methods	Reported outcomes
1	Mapping capacity building programs in health diplomacy: Relevance and application in an uncertain world	Pattanshetty et al. (13), 2023	Curriculum/programs mapping (review of 50 global initiatives)	Students, professionals, diplomats	Varied (short courses to full programs)	Mainly in-person	Mixed methods (lectures, negotiation exercises, cross-cutting themes)	Governance, international relations, law, public policy, crisis management, cross-cultural negotiation	Literature synthesis	Identified dominance of Global North, need for inclusion of economics, politics, and environment
2	Key competencies and training framework for health diplomacy: a guidance document	GHDIN (15), 2025	Framework/guidance for training	Practitioners, diplomats, health professionals	Flexible (not prescriptive)	Blended/adaptable	Competency-based, peer learning, intergenerational exchange	Global health systems, diplomacy, decision-making, cross-cultural communication, advocacy	Competency mapping (not empirical evaluation)	Proposed structured competency framework for institutional adoption
3	Navigating global health diplomacy: challenges and opportunities in building a community of practice	Rosenbaum et al. (12), 2025	Qualitative interviews (capacity-building exploration)	Experienced GHD practitioners (*n* = 54, across 23 countries)	N/A (experiential, professional setting)	Professional practice/informal	Situated learning, networks, peer knowledge transfer	Policy analysis, negotiation, leadership, tacit knowledge sharing, network integration	Thematic qualitative analysis (interviews)	Identified challenges in knowledge transfer, leadership gaps, opportunities for stronger communities of practice
4	American Mock World Health Organization: an Innovative Model for Student Engagement in Global Health Policy	Lei et al. (6), 2017	Student conference/simulation	Students (undergraduate/graduate)	3-day simulation conference	In-person, role-play	Experiential, simulation of WHA debates, stakeholder negotiation	Diplomacy, negotiation, public speaking, policy drafting, conflict resolution	Post-conference surveys	90–98% satisfaction, perceived paradigm shift, career influence reported
5	Building capacity and capability for science diplomacy: challenges in decolonizing the curriculum for Global Health System Leadership	Millar et al. (7), 2025	Academic program (Global Health System Leadership, IBC)	Students (UoB Dubai, postgraduate)	Semester/degree-based	Blended (branch campus model)	Formal courses, leadership development, reflexive/decolonial pedagogy	Leadership, systems thinking, diplomacy, critical inquiry, reflexivity	Case study reflection	Highlighted decolonization challenges, knowledge exchange limitations, local–global tensions
6	Applied global health diplomacy: profile of health diplomats accredited to the United States and foreign governments	Brown et al. (11), 2018	Professional training need assessment	Health Attachés (US + foreign)	Ongoing career training	On-the-job, mentorship	Interviews, reflective practice	Diplomacy, negotiation, applied science, cross-cultural competence	Qualitative interviews (*n* = 7)	Identified need for structured career pathways, competencies with mastery levels, mentorship
7	Developing global health diplomacy-related skills using a COVID-19-like epidemic simulation as a learning strategy	Ortiz et al. (8), 2021	Scenario-based training (problem-based learning)	MPH students (USF)	One classroom session (early semester)	In-person simulation	Problem-based learning, interdisciplinary, cultural briefings	Diplomacy, negotiation, communication, crisis response	Pre-post surveys, focus group	Significant self-reported improvement in diplomacy, negotiation, emergency response skills
8	Capacity building on health diplomacy: a training experience from Pakistan	Shaikh et al. (14), 2018	National training course (capacity building workshop)	Mid- and senior-level officials, health professionals, diplomats, academics	Short-term workshop (multi-day)	In-person	Lectures, panel discussions, case studies, group work, SDG-focused conference	Negotiation, governance, cross-sectoral collaboration, human rights, health security, systems thinking	Descriptive feedback (no formal pre/post assessment due to seniority of participants)	Enhanced knowledge of foreign policy-health linkages, improved understanding of diplomacy in health; replicable model for other EMR countries

### 2.5 Collating, summarizing, and reporting the results

We synthesized the extracted data using a combination of descriptive statistics and narrative synthesis. We calculated frequencies and distributions to provide an overview of the types of initiatives, delivery modes, learning outcomes and competencies reported across the included studies. We mapped training initiatives according to their geographic regions, target audiences, and skill domains, allowing us to identify patterns and contextual variations in health diplomacy education. In addition, we conducted a qualitative thematic analysis to identify key gaps and recurring insights across the literature. To enhance clarity and accessibility, we presented the findings using tables and charts that visually summarized the distribution of initiatives and highlighted emerging trends.

## 3 Results

**Figure 2:**
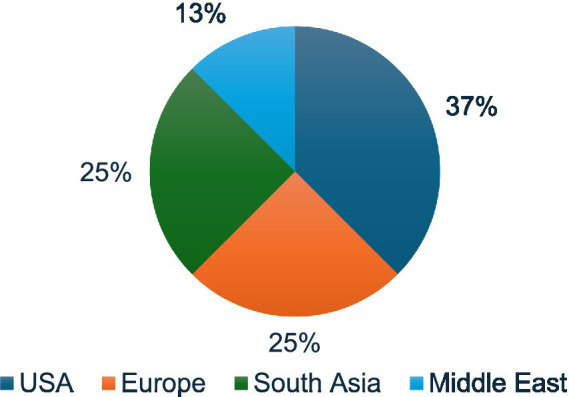
Geographic distribution of initiatives.

We identified a total of eight initiatives and frameworks across the included literature, spanning from 2017 to 2025. These initiatives represented a range of educational formats, including student simulations, academic programs, professional training needs assessments, curriculum frameworks, and competency-based guidance documents. The majority of initiatives originated from the Global North with fewer examples from the Middle East and South Asia (Figure 2).

### 3.1 Geographic distribution

Most initiatives originated from the United States (*n* = 3), followed by Europe (*n* = 2), South Asia (*n* = 2), and the Middle East (*n* = 1). The overall landscape reflects the dominance of Global North institutions in defining and advancing global health diplomacy (GHD) education.

### 3.2 Target audiences

The training initiatives identified in this review catered to a wide spectrum of learners, reflecting the diverse skill demands in health diplomacy.

Students represented a major target group, with three studies focusing on undergraduate, graduate, or postgraduate learners. Examples include the American Mock WHO Conference (6), where students practice negotiation and diplomacy through simulated World Health Assembly sessions, and the epidemic simulation activity at the University of South Florida (8), which introduced MPH students to crisis negotiation and communication in a classroom setting. Additionally, Millar et al. (7) described a semester-long postgraduate program in Global Health Leadership at the University of Birmingham Dubai, aimed at embedding diplomacy and systems thinking within formal academic curricula.

Professionals and practitioners were the focus of another four studies, emphasizing the training of diplomats and health attachés and senior officials in active practice of health diplomacy. Brown et al. (11) highlighted the need for structured career pathways and mentorship for health attachés, while Rosenbaum et al. (12) examined capacity-building and network development among experienced global health diplomats across 23 countries. Pattanshetty et al. (13) mapped existing health diplomacy curricula, many of which targeted professional learners seeking to expand expertise in governance, negotiation, and international policy. Similarly, Shaikh et al. (14) reported on a capacity-building workshop in Pakistan, which trained mid- and senior-level officials, diplomats, and professionals from multiple sectors through a contextualized curriculum that included negotiation, governance, and cross-sectoral collaboration.

Finally, we identified mixed audiences addressed by multiple adaptable frameworks. The Global Health Diplomacy Network’s Training Framework (2025) was designed to be flexible, allowing institutions to tailor training for both students and practicing professionals (15). This adaptability highlights the recognition that GHD requires collaboration across experience levels, blending fresh academic perspectives with practitioner expertise (see Table 2).

**Table 2:** Target audiences of training initiatives.

Target audience	Number of studies	Study IDs	Examples of initiatives
Students (undergraduate/graduate/postgraduate)	3	(4, 5, 7)	American Mock WHO (student conference), COVID-19 epidemic simulation (MPH classroom), Global Health Leadership program (postgraduate, UoB Dubai)
Professionals and practitioners (diplomats, health attachés, experienced GHD practitioners)	4	(1, 3, 6, 8)	Health attaché training needs assessment, community of practice exploration, curriculum mapping for professional programs, national training workshop
Mixed audiences (flexible participation by students and professionals)	1	(2)	Global Health Diplomacy Training Framework (adaptable for diplomats, health professionals, and trainees)

### 3.3 Types and duration of initiatives

The initiatives identified in this review varied considerably in both their format and duration, ranging from short, intensive experiences, to career-long professional development pathways.

Short-term training opportunities were the most common, providing concentrated exposure to GHD skills within highly interactive learning environments. Examples included single classroom simulations, such as the COVID-19 epidemic scenario developed by Ortiz et al. (8), which introduced students to negotiation and crisis response skills within the span of a single academic session. Similarly, the American Mock World Health Organization (AMWHO) described by Lei et al. (6) offered an immersive three-day conference where student delegates engaged in role-play simulations of a session of the World Health Assembly. In South Asia, Shaikh et al. (14) reported on a multi-day national training workshop in Pakistan, which trained mid- and senior-level officials from across sectors using lectures, case studies, panel discussions, and group work. These short-term models emphasized experiential and participatory learning, providing both students and professionals with opportunities to practice diplomacy and negotiation in condensed but contextually rich formats.

At the other end of the spectrum, we identified medium- to long-term initiatives, notably the Global Health System Leadership program delivered at the University of Birmingham Dubai (7). This degree-based curriculum extended over a semester or more and was embedded within broader postgraduate education. Unlike short simulations, these longer programs sought to integrate global health diplomacy into a more comprehensive leadership framework, blending coursework with reflexive and decolonial pedagogical approaches (7). In addition to structured academic offerings, some initiatives were designed as flexible frameworks rather than time-bound courses. The Global Health Diplomacy Network’s (GHDIN) Training Framework (2025) exemplifies this category, providing a competency-based model that institutions and organizations can adapt to their own contexts. Such frameworks allow for modular learning and are not tied to fixed timelines, offering adaptability across professional and educational settings (15).

Finally, we identified career-long training initiatives identified in the form of professional pathways for health attachés (11). Unlike discrete courses or programs, these initiatives emphasized continuous learning through on-the-job experiences, mentorship, and reflective practice (11). This approach recognizes that global health diplomacy is a competency based professional pursuit, with some skills requiring significant investments of time for practitioners to develop, refine and strengthen skills throughout their diplomatic careers.

These findings illustrate a spectrum of training opportunities (ranging from short, intensive simulations, to ongoing professional development) reflecting the diverse needs of students, professionals, and career diplomats in the evolving field of global health diplomacy.

### 3.4 Delivery modes and pedagogical approaches

Across the included studies, the delivery of training initiatives was predominantly delivered in-person, particularly in student-centered simulations and professional development programs. Conferences such as the American Mock WHO (6) and classroom-based activities like the COVID-19 epidemic simulation (8) relied heavily on face-to-face interaction to recreate authentic negotiation and decision-making environments. However, some programs adopted blended or adaptable models, particularly within academic institutions and international frameworks, which allowed for tailoring to diverse audiences and contexts (7, 15).

In terms of pedagogy, simulation-based experiential learning emerged as a cornerstone approach. The AMWHO model engaged students through mock World Health Assembly debates, role-play, and resolution drafting to build practical skills in diplomacy and negotiation (6). Similarly, the epidemic simulation exercise emphasized scenario-based crisis response, requiring students to apply knowledge in real-time decision-making environments (8).

Beyond simulations, several initiatives incorporated competency-based and problem-based learning approaches. For instance, the COVID-19 epidemic simulation was explicitly grounded in problem-based learning and Bloom’s taxonomy, ensuring that students progressed from knowledge acquisition to applied critical thinking (8). Likewise, the GHDIN framework emphasized competency mapping and measurable learning outcomes to guide training design (15). The Pakistan workshop also used case studies and panel-led discussions, offering applied learning opportunities while contextualizing global diplomacy concepts within national and regional realities (14).

Professional training and community-of-practice initiatives further highlighted the importance of peer-to-peer and intergenerational knowledge exchange. Rosenbaum et al. (12) found that seasoned diplomats and practitioners valued mentorship, reflective practice, and tacit knowledge transfer as critical mechanisms for building sustainable capacity in global health diplomacy. Similarly, Brown et al. (11) emphasized the role of mentorship and structured career pathways for health attachés, underscoring that learning in this domain extends beyond classrooms into long-term professional practice. Academic programs at international branch campuses, such as the Global Health System Leadership course at the University of Birmingham Dubai have adopted formal coursework combined with reflexive and decolonial pedagogies (7). These approaches recognized the importance of critically examining power dynamics, encouraging students to reflect on global–local intersections in health diplomacy, and equipping them with contextually relevant leadership skills.

These delivery modes and pedagogical strategies highlight a spectrum of approaches, from immersive simulations and national workshops to reflexive academic curricula, all aimed at equipping learners with the knowledge and competencies necessary for effective global health diplomacy.

### 3.5 Competencies and skill domains addressed

Across the reviewed initiatives, negotiation and diplomacy skills were the most consistently addressed, appearing in all eight studies. Whether through structured simulations, such as the American Mock WHO (6), problem-based epidemic scenarios (8), national-level training like the Pakistan capacity-building workshop (14) or professional practice among health attachés (11), the centrality of negotiation reflects a core role that diplomacy plays in global health. Institutional frameworks, including the GHDIN competencies, further reinforce diplomacy as a foundational skill for both students and practitioners (15).

We identified cross-cultural communication and leadership emphasized in six initiatives, recognizing that effective diplomacy requires navigating diverse cultural contexts while also demonstrating leadership capacity. The profession of Health attachés underscored the importance of intercultural skills for advancing negotiations across borders (11), while frameworks such as the GHDIN guidance (15) highlighted coalition-building and leadership as key competencies. Similarly, the University of Birmingham Dubai program incorporated leadership and reflexive practice as central learning outcomes (7). The Pakistan workshop also emphasized cross-sectoral collaboration and governance, underscoring the importance of intercultural and interprofessional dialogue for advancing health diplomacy in complex national and regional contexts (14) (see Table 3).

**Table 3:** Competencies and skill domains in training initiatives.

Competency/skill domain	Number of studies	Study IDs	Examples of initiatives
Negotiation and diplomacy skills	8	(1, 2, 3, 4, 5, 6, 7, 8)	Mock WHO simulation, epidemic crisis negotiation, attaché training, competency framework, CoP peer learning, curriculum reviews, academic leadership programs
Cross-cultural communication and leadership	6	(2, 3, 4, 5, 6, 8)	Attaché intercultural skills, global framework competencies, peer network exchanges, WHA role-play, postgraduate leadership program
Policy analysis and governance knowledge	5	(1, 2, 3, 5, 8)	Mapping of curricula (policy emphasis), governance frameworks, practitioner networks, academic courses
Crisis management and emergency response	3	(1, 6, 7)	Epidemic simulation, attaché crisis response, mapped training programs
Advocacy, coalition-building, and systems thinking	4	(2, 3, 5, 8)	Coalition-building competencies, community of practice learning, systems leadership programs

Policy analysis and governance knowledge featured in four studies, particularly in training programs aimed at equipping professionals with the ability to understand and influence health policy structures. Curriculum mapping exercises revealed a strong emphasis on governance and systems knowledge (13), while the GHDIN framework provided structured pathways to connect governance with negotiation (15). Professional networks and academic programs also underscored the importance of linking policy analysis to leadership (7, 12).

We also found crisis management and emergency response competencies in three initiatives, reflecting a focus on case studies that identified lessons learned from health crises such as COVID-19 and Ebola. The epidemic simulation directly targeted students’ ability to negotiate and communicate during outbreak response scenarios (8), while health attachés highlighted the importance of crisis preparedness in diplomatic practice (11). Mapping studies also identified crisis management as a recurring but underdeveloped domain (13).

Advocacy, coalition-building, and systems thinking appeared in three initiatives, reflecting a growing recognition of the need for collaborative approaches to global health. The GHDIN framework explicitly outlined coalition-building as a core competency (15), while communities of practice emphasized advocacy through networks and tacit knowledge sharing (12). Academic programs such as the Global Health Leadership course integrated systems thinking and critical inquiry into their curricula, aiming to build leaders capable of navigating complex intersectoral challenges (7).

These findings illustrate that while negotiation remains a central skill domain across all initiatives, there is increasing attention to leadership, cross-cultural competence, and systems-level approaches that broaden the scope of global health diplomacy training.

### 3.6 Evaluation and outcomes

Evaluation approaches across the identified initiatives were diverse but limited to short-term, self-reported outcomes, reflecting a gap in long-term impact assessments. Several studies relied on post-activity surveys to measure participant satisfaction and perceived learning. For instance, the American Mock WHO simulation reported very high approval, with 90%–98% of participants rating the experience as good or better, and many indicating that it influenced their career trajectories (6). Similarly, other student-centered workshops and conferences used structured surveys to capture immediate feedback, with participants consistently reporting improvements in diplomacy, negotiation, and communication skills. A smaller number of initiatives employed pre- and post-designs to assess changes in identified skills. The COVID-19 epidemic simulation conducted among MPH students demonstrated significant self-reported gains in understanding diplomacy, negotiation, and public health emergency response, with statistical improvements confirmed through mixed-methods evaluation (8). These findings highlight the potential of scenario-based, problem-centered learning to produce measurable short-term outcomes in student populations.

Among professional audiences, qualitative interviews with health attachés and practitioners provided rich insights into training needs and skill development gaps. Brown et al. (11) emphasized that while attachés recognized the importance of diplomacy, negotiation, and cross-cultural competence, they also identified the absence of structured career pathways, mentorship opportunities, and defined competency mastery levels. These perspectives underscored the necessity of moving beyond *ad hoc* learning toward more systematic professional development in global health diplomacy. Likewise, Rosenbaum et al. (12) reported that communities of practice valued mentorship and peer learning but noted challenges in knowledge transfer and leadership development. In the Pakistan capacity-building workshop, Shaikh et al. (14) reported participant feedback that indicated improved understanding of global health diplomacy concepts, stronger appreciation of foreign policy linkages, and enhanced confidence in cross-sectoral collaboration. However, similar to other initiatives targeting senior officials, no formal pre- and post-assessment was conducted, reflecting a common limitation in evaluating professional training programs.

Some initiatives, particularly framework-driven approaches, have not report empirical evaluation results at all. The GHDIN competency framework (2025) provided structured benchmarks and guidance for institutions, but lacked outcome-based assessment of effectiveness (15). Similarly, curriculum mapping studies synthesized program content, but did not track learner outcomes (13).

The evaluations demonstrate strong short-term evidence of learner satisfaction and self-reported skill development, but highlight a need for rigorous, longitudinal assessments translates into sustained institutional strengthening or improved diplomatic outcomes in global health. Further, learning institutions are encouraged to adopt the competency-based education approach, which aligns learning inputs and outcomes to learner measurement and into labor market recruitment, performance assessment and career advancement.

### 3.7 Assessment of gaps in GHD initiatives

#### 3.7.1 Gaps in evaluation

While the reviewed initiatives show innovations in teaching global health diplomacy, we similarly identified important gaps in evaluation. Most studies relied on self-reported outcomes immediately following training, such as satisfaction surveys (6) or short-term pre- and post-activity assessments (8). These measures offer useful insights into learner perceptions, but do not capture competencies retained, applied, or translated into professional practice over time. Additionally, evaluations lacked standardized outcome metrics. Studies varied widely in how they defined and measured success, ranging from subjective participant ratings to thematic analysis of interviews (11, 12), making it difficult to compare results across initiatives or build a cohesive evidence base for best practices. Similarly, in the Pakistan workshop (14), participant feedback highlighted improved understanding of health diplomacy and cross-sectoral collaboration, but the absence of formal pre/post evaluation limited systematic assessment of impact.

Another gap is the absence of longitudinal or institutional-level evaluations. Few initiatives tracked whether skill gains influenced career progression, policy engagement, or institutional capacity overall. For example, although attachés emphasized the importance of structured career pathways (11), no studies evaluated whether training programs supported advancement or improved global health action or outcomes. Similarly, frameworks such as the GHDIN competency model (2025) offered structured guidance but did not report on implementation outcomes (15). Finally, there was little evidence of comparative or cross-contextual evaluation. Programs implemented in different regions (e.g., United States, Europe, South Asia, Middle East) operated in diverse political and institutional environments, yet few studies systematically examined how context shaped effectiveness or transferability of training. These gaps underscore the need for more rigorous, standardized, and longitudinal evaluation designs to illustrate effectiveness of global health diplomacy training and to ensure that acquired competencies contribute to sustainable policy, program, and leadership outcomes.

#### 3.7.2 Gaps in training

A major gap identified in the review was the underrepresentation of Global South leadership in global health diplomacy (GHD) training. Most initiatives are designed and implemented by institutions in the United States and Europe, with comparatively few South-led or regionally driven programs. While some efforts, such as the University of Birmingham Dubai program (7), attempted to situate training in non-Western contexts, these remained largely driven by institutions in the Global North. An exception was the Pakistan capacity-building workshop (14), which was locally led and regionally contextualized, yet such examples remain limited. This imbalance reflects broader structural inequities in global health education and highlights the need for more inclusive, locally led initiatives (13). This brings into question the applicability and relevance of many of the available programs to the cultural and political milieu of the global south.

Another recurring gap was related to evaluation limitations. Most initiatives employed short-term, self-reported outcomes such as satisfaction surveys (6) or pre- and post-assessments of perceived skill improvement (8). While valuable for immediate pedagogical feedback, these evaluations did not capture whether competencies are retained or applied in practice. Only qualitative studies with health attachés (11) and professional practitioners (12) provided insights into longer-term challenges, although these were not systematically measured. Similarly, the Pakistan workshop relied on participant feedback rather than structured pre- and post-evaluation evaluation, limiting evidence of its sustained impact (14).

The training landscape showed considerable fragmentation. Initiatives differed widely in their scope, pedagogical approaches, competencies addressed, and evaluation methods. This diversity reflects innovation and context sensitivity but also makes it difficult to establish common benchmarks or to compare outcomes across studies.

A further gap can be the failure to design curricula and learning outcomes to practice and practice-based learning. Graduates, as well as continuous learnings, should be supported to gain knowledge, skills and competencies that are sought and measured within the labor market. Some of the examples in this research engage target employers within simulation and practice activities designed to build competencies. Engaging these organizations’ human resources specialists could further link learner capacities with recruitment and job definition. Further, dedicated engagement with employers of public health diplomats offers a feedback mechanism to validate and strengthen program design.

### 3.8 Key insights from scoping review

Despite these gaps, we observed several important insights across the initiatives.

First, communities of practice emerged as vital to sustaining GHD capacity. Professional networks, mentorship, and tacit knowledge-sharing are consistently highlighted as mechanisms that complemented formal training. For example, Rosenbaum et al. (12) underscored the importance of peer-to-peer knowledge exchange in strengthening diplomatic skills, while Brown et al. (11) emphasized mentorship and on-the-job learning as critical for health attachés.

Second, there was a strong call for decolonization of curricula, and yet few if any emphasized the interface between local culture and politics on global health strategy and action. Training initiatives situated in the Middle East have demonstrated the importance of reflexive and context-sensitive pedagogies that acknowledge and address colonial legacies in global health education (7). This insight reflects a broader push for curricula that are more inclusive, locally relevant, and critically engaged with power dynamics in global health.

Finally, the review identified the importance of career pathways in maturing GHD as a professional discipline. Health attachés highlighted the lack of structured career progression, competency mastery frameworks, and mentorship opportunities to support sustained professional development (11). Without clear pathways, training risks being episodic rather than contributing to long-term institutional and career-level capacity building, needed to bring about global health action.

These insights underscore the need for global health diplomacy training that extends beyond short-term skill acquisition to encompass sustained professional networks, decolonial approaches to curriculum design, and structured career pathways (see Table 4).

**Table 4:** Gaps and key insights in health diplomacy training.

Category	Theme	Description/evidence	Supporting sources (study IDs)
Gaps	Underrepresentation of Global South	Training initiatives predominantly designed and led by Global North institutions; limited South-led leadership or authorship in curriculum design.	(1, 5, 8)
	Evaluation limitations	Few programs assessed long-term effects on career trajectories, institutional capacity, or policy impact; reliance on short-term, self-reported surveys.	(4, 6, 7, 8)
	Fragmentation	Programs varied widely in scope, pedagogy, competencies addressed, and outcome measures, limiting comparability and standard-setting.	(2, 3)
Key insights	Communities of practice	Peer learning, mentorship, and tacit knowledge exchange emphasized as crucial for sustainable capacity building.	(3, 6)
	Decolonization of curricula	Recognition of colonial legacies in curricula; calls for reflexive, context-sensitive approaches in training, especially at international branch campuses.	(5)
	Career pathways	Health attachés highlighted the lack of structured career progression, mentorship, and mastery frameworks in GHD professional development.	(6)

### 3.9 Linking health diplomacy training to public health practice

The findings of this review demonstrate that training initiatives in global health diplomacy (GHD) are integral to the broader practice of public health diplomacy (PHD), which operates at the interface of health and international relations. Public health diplomacy is defined as a multidisciplinary field that equips practitioners to communicate, facilitate, negotiate, and build consensus using systems thinking and evidence-based, community-informed approaches. It is grounded in equity-focused and human-centered values, with the ultimate goal of improving health and well-being for all (16). The emphasis on competencies such as negotiation, cross-cultural communication, leadership, and crisis management reflects the practical skills required for Public Health Diplomacy practitioners, applicable to both the domestic and global roles, who must navigate complex multilateral environments with often competing interests. Initiatives like the American Mock WHO simulation (6) and epidemic response exercises (8) replicate the decision-making contexts in which health and foreign policy intersect, preparing students to apply diplomacy in real-world crises. Similarly, professional training needs identified by health attachés underscore that PHD extends beyond technical knowledge, requiring structured mentorship, tacit knowledge exchange, and career-long development to support practitioners in influencing health policy and program outcomes at global and national levels (11).

At the same time, the gaps and insights identified across the reviewed initiatives reveal challenges and opportunities for strengthening public health diplomacy. The dominance of Global North institutions in shaping curricula reflects ongoing power asymmetries in global health education (13), while limited evaluation methods and fragmented program designs hinder the establishment of shared standards. Yet, several promising directions emerge: the importance of communities of practice in sustaining capacity (12), the call for decolonizing curricula to better reflect diverse contexts and histories (7), and the need for structured career pathways to institutionalize GHD as a professional discipline (11). Together, these insights suggest that effective training is not only about equipping individuals with discrete skills but also about transforming institutions and systems to strengthen the practice of public health diplomacy in an interconnected and inequitable world.

## 4 Discussion

This scoping review mapped the landscape of health diplomacy education, highlighting the diversity of curricula, training approaches, and competency frameworks developed in the past decade. The findings reveal that while there is a broad spectrum of initiatives—from short-term student simulations to professional development and institutional frameworks—all share a common aim: to equip learners with the skills required to advance health objectives in complex diplomatic contexts, balancing competing interests. Results reinforce the conceptualization of public health diplomacy as a practice situated at the intersection of health and international relations, requiring both technical knowledge and diplomatic acumen.

We addressed negotiation and diplomacy skills in all initiatives reviewed, confirming their central role in PHD practice, both locally and globally. Student-focused programs such as the American Mock WHO (6) and the COVID-19 epidemic simulation (8) prioritized negotiation, communication, and decision-making under crisis, aligning closely with the practical demands of multilateral diplomacy. Professional training, including the health attaché competency assessment (11), emphasized not only negotiation but also leadership and cross-cultural competence, skills required for navigating international health governance structures. Similarly, curriculum mapping exercises demonstrated that governance, policy analysis, and law are consistently integrated into global health diplomacy education (13). These findings mirror broader competency frameworks proposed for global health leadership, which highlight systems thinking and negotiation as indispensable skills (7).

The initiatives revealed a strong trend toward experiential and competency-based pedagogy. Simulations and problem-based learning approaches allowed students to engage with complex scenarios that mimic real-world diplomatic challenges (6, 8). These findings align with trends in medical and public health education, where scenario-based learning demonstrated to enhance critical thinking and informed decision-making. Professional and practitioner-focused programs further highlighted the role of peer-to-peer and intergenerational knowledge transfer, where communities of practice were identified as essential for sustaining capacity (12). This resonates with the broader literature on policy learning, which emphasizes networks as key vehicles for the diffusion of knowledge. These competencies are also consistent with the findings of Joshi et al., who discussed a range of training needs, skills, and knowledge areas required for public health professionals to effectively serve as advocates in public health diplomacy (16). When designing learning and curricula for health practitioners, including health diplomats, it is important to consider the Global Competency Framework for Universal Health Coverage (17). With this framework, WHO sets out its recommended approach to competency-based health worker education outcomes; in so doing, it also provides conceptual and terminological clarity. The six domains of health worker competencies towards the achievement of UHC are relevant to the practice of health diplomacy: people-centeredness, decision-making, communication, collaboration, evidence-informed practice and personal conduct.

Despite pedagogical innovation, the evaluation of health diplomacy training remains underdeveloped. Student initiatives such as AMWHO relied heavily on satisfaction surveys, reporting very high approval rates (90%–98%) and positive perceptions of skill development (6). Similarly, Ortiz et al. (8) demonstrated statistically significant gains in self-reported competencies through a pre/post evaluation. While encouraging, these self-reported, short-term outcomes are insufficient to assess whether competencies are retained or applied in professional practice. Professional perspectives highlight the same limitation: health attachés identified gaps in structured career pathways and competency mastery but provided little evidence of systematic training evaluation (11). These findings are consistent with critiques in broader global health education literature, where outcome assessments tend to privilege short-term learner feedback over longitudinal career or policy impact.

Three key gaps emerged from the evidence. First, underrepresentation of the Global South was evident, with most programs led by institutions from the Global North. Even when training was situated in the Middle East, such as the University of Birmingham Dubai program, program design remained externally driven (7). This reflects wider inequities in global health governance and echoes calls for South-led leadership in training and agenda setting (13). While most identified initiatives were developed and implemented in Global North contexts, the transferability of these models to other regions remains uncertain. Training programs grounded in Western governance systems, pedagogical norms, and policy frameworks may not align with the institutional realities, diplomatic cultures, or health priorities of the Global South. These contextual differences highlight the complexities of transferring curricula across regions, reinforcing the need for co-created, locally led approaches that adapt content and pedagogy to regional contexts rather than replicating Northern models. Second, evaluation limitations persisted across studies, as few programs systematically tracked career trajectories or institutional outcomes. Finally, the training landscape remains fragmented, with heterogeneity in scope, pedagogy, and evaluation limiting comparability across initiatives (12, 15).

Our findings complement and extend previous studies on health diplomacy training and competencies. For example, Pattanshetty et al. (13) broadly mapped global capacity-building programs but did not systematically assess pedagogical approaches or evaluation methods. Brown et al. (11) focused on the perspectives of health attachés, identifying essential competencies such as negotiation and cross-cultural communication, yet without analyzing how such skills are taught within formal training programs. Similarly, Rosenbaum et al. (12) emphasized the role of communities of practice and tacit knowledge-sharing in building diplomatic capacity, but their study did not examine structured curricula. In contrast, this review systematically synthesizes evidence on health diplomacy education between 2015 and 2025, with attention to curricula, teaching methods, competencies, and evaluation, thereby bridging practitioner-identified needs with formal educational responses and highlighting opportunities to strengthen public health diplomacy through education. Further, there may also be a need to better map these core competencies to the hierarchical domains of GHD to make them more practitioner specific, such identifying how competencies lead to professional development for core, multistakeholder, and informal diplomacy actors (2).

Further, several health diplomacy focused programs and educational offerings were not included in our analysis due to our focus on GHD education and training that were discussed or evaluated in the literature. These include long-standing GHD executive programs (e.g., Graduate Institute of Geneva, University of Toronto executive programs), other GHD courses as part of formal university curriculum and offered for credit (e.g., University of Geneva,), online course offerings (e.g., Coursera GHD course offered by SUNY), and professional certificate programs (e.g., Georgetown University Graduate Certificate). Further, GHD curriculum may be imbedded in other public health or global health course offerings, and a comprehensive course or syllabus review was not conducted to identify these offerings. Review of these additional GHD education and training opportunities may provide more insights into pedagogical approaches, geographic distribution, competencies taught, and relevant evaluation and outcomes.

Comparative insights from global health education suggest that embedding competency-based frameworks, integrating mentorship, and strengthening South-led initiatives are essential next steps. Ultimately, for PHD to mature as a discipline, education and training must move beyond episodic workshops or student simulations to become sustained, institutionalized, and globally inclusive pathways that equip practitioners to meet the health challenges of an interconnected world.

### 4.1 Strengths and limitations

This review has several notable strengths. By systematically mapping curricula, training approaches, and competency domains, it provides a comprehensive overview of the emerging field of health diplomacy education. The inclusion of diverse sources, spanning student-focused simulations, academic degree programs, professional development pathways, and competency frameworks, enabled a broad synthesis that captures both the diversity and commonalities across these initiatives. The review also highlights cross-cutting competencies, pedagogical innovations, and structural gaps, offering a foundation for future curriculum design and policy development. Importantly, by linking findings explicitly to the broader concept of public health diplomacy, this review contributes to bridging the gap between training initiatives and their implications for practice and governance. The review brings to light that GHD training does not generally emphasize the applicability of diplomacy skills to local/domestic consensus building, policy making, strategy building and implementation.

However, several limitations must be acknowledged. First, the review was limited to available published literature, which may exclude relevant but unpublished training initiatives. For example, several health diplomacy focused programs and educational offerings were not included in our analysis due to our focus on GHD education and training that were discussed or evaluated in the literature. Second, heterogeneity in study designs, evaluation methods, and reporting limited the ability to conduct comparative analysis or meta-synthesis. Many initiatives relied on descriptive accounts or short-term self-reported outcomes, making it difficult to assess long-term effectiveness. Third, the review did not include quantitative meta-analysis due to the absence of standardized evaluation metrics across studies. Finally, while the scoping methodology enabled a broad mapping of the field, it does not allow for detailed assessment of intervention effectiveness, which would require future systematic reviews with more stringent inclusion criteria.

### 4.2 Recommendations

Based on the findings of this review, this research draws several recommendations to strengthen health diplomacy education and, by extension, the practice of public health diplomacy. First, there is a need to institutionalize competency-based frameworks that standardize key skills such as negotiation, leadership, and cross-cultural communication while allowing flexibility for local adaptation. Second, programs should prioritize longitudinal evaluation strategies that go beyond short-term surveys to assess the retention of competencies, their application in practice, and their influence on career trajectories and institutional capacity, as well as assess impact on public health programs and initiatives. Third, training should be embedded within communities of practice and mentorship structures, recognizing that diplomatic competencies are refined over time through networks, peer exchange, and lived professional experience. Finally, the decolonization of curricula should be an explicit objective, particularly in international branch campuses and global partnerships, to ensure that pedagogy is reflexive, inclusive, and contextually relevant.

## 5 Conclusion

This scoping review mapped the current landscape of health diplomacy education, identifying a diverse set of initiatives ranging from short-term simulations and academic programs to professional development frameworks and career-long learning pathways. Across these efforts, negotiation, leadership, cross-cultural communication, and policy analysis emerged as core competencies, reflecting the essential skillset required for effective public health diplomacy. While innovative pedagogical approaches such as simulations, problem-based learning, and reflexive curricula demonstrate promising ways to prepare learners, significant gaps remain, particularly in evaluation methods.

The review highlights the need for training that not only equips individuals with technical skills but also fosters sustained professional pathways, institutional capacity, and inclusive approaches to curriculum design. There is a pressing need to integrate public health diplomacy training into the broader discipline of public health education. Embedding research on teaching and learning in this area will help build an evidence base for what approaches most effectively strengthen health diplomacy practice and global health governance. Strengthening health diplomacy education is therefore critical to advancing public health diplomacy as a professional discipline capable of addressing global health challenges in an interconnected and inequitable world.
