# Supplementary material for: Health diplomacy training, pedagogical approaches, and skills assessment: a scoping review
Source: Front Public Health. 2025 Dec 16;13:1729728. doi: 10.3389/fpubh.2025.1729728 (PMC12755105; doi:10.3389/fpubh.2025.1729728)
Supplement: Supplementary file 1 [file Table_1.docx]

**Supplement 1. Search terms used in this study**

| **Search number** | **Query** | **Filters** | **Results** |
| --- | --- | --- | --- |
| **1** | health diplomacy curriculum | in the last 10 years, Free full text, English, Exclude preprints | 83 |
| **2** | health diplomacy skills | in the last 10 years, Free full text, English, Exclude preprints | 24 |
| **3** | health diplomacy education | in the last 10 years, Free full text, English, Exclude preprints | 99 |
| **4** | health diplomacy competency | in the last 10 years, Free full text, English, Exclude preprints | 149 |
| **5** | health diplomacy training | in the last 10 years, Free full text, English, Exclude preprints | 112 |
| **6** | global health diplomacy training | in the last 10 years, Free full text, English, Exclude preprints | 92 |
| **7** | "global health diplomacy training" | in the last 10 years, Free full text, English, Exclude preprints | 92 |
| **8** | "global health diplomacy skills" | in the last 10 years, Free full text, English, Exclude preprints | 15 |
| **9** | global health diplomacy education | in the last 10 years, Free full text, English, Exclude preprints | 84 |
| **10** | global health diplomacy curriculum | in the last 10 years, Free full text, English, Exclude preprints | 70 |
| **11** | global health diplomacy competency | in the last 10 years, Free full text, English, Exclude preprints | 133 |
